# How backers’ behavior affects financing performance in agri-food reward-based crowdfunding: The moderating mechanism of initiator characteristics and project attributes

**DOI:** 10.1371/journal.pone.0305752

**Published:** 2024-07-05

**Authors:** Junjuan Du

**Affiliations:** College of Economics & Management, Hefei Normal University, Hefei, Anhui, China; Palestine Technical University - Kadoorie, STATE OF PALESTINE

## Abstract

Crowdfunding is a new type of financing favored by entrepreneurs in need of capital. Financing performance is a key concern for crowdfunding project initiators. Although a growing number of studies have investigated the factors that affect the financing performance of crowdfunding projects, there are still some issues that need to be further clarified. How does the investment behavior of backers, as the supply side of finance, affect the financing performance of project in reward-based crowdfunding? What are the moderating mechanisms of this influence by initiator characteristics and project attributes? Based on a panel data set from Zhongchou, a famous agri-food crowdfunding platform in China, this paper finds that the investment speed, the investment intensity, the number of early backers, the backers’ comments, and the number of selfless backers all have significant effects on financing performance. The core trust factors of initiator characteristics and project attributes play a moderating role in the relationship between backer investment behavior and financing performance, but there are differences in the moderating mechanisms. Based on the research conclusions, practical enlightenment is proposed for initiators, crowdfunding platforms, and regulators.

## Introduction

Early-stage ventures, in particular, encounter challenges in obtaining long-term external financing because it is difficult and expensive for them to raise capital from banks and investors. Thus, smaller and younger firms often encounter liquidity constraints from this lack of outside financing [1]. Crowdfunding is a new way to acquire capital via the internet, allowing entrepreneurs to solicit funds from the “crowd” for innovative and new projects [2]. The preference of entrepreneurs in favor of crowdfunding is stronger than that of venture capital [3–5]. The reward-based crowdfunding, one type of crowdfunding, usually provides the product or service or non-monetary reward to the backers, who are customers of the product or service [6]. Agri-food crowdfunding is a reward-based crowdfunding in agriculture, essentially a pre-sale of agricultural products that solves the dual challenges of marketing and financing in agricultural production. Due to the unique geographical, seasonal and cyclical nature of agricultural production, agri-food crowdfunding projects need to be financed in the shortest possible time, also ensuring that backers get fresh produce as early as possible. Engaging the active participation of backers is the key to the rapid completion of the project and has the following important implications. First, the active participation of backers can reflect the popularity and market recognition of agricultural products in crowdfunding. The cultivation of potential backers in the early stage of the project can help the initiator to keep abreast of consumer demand and provide support to start the next round of crowdfunding as soon as possible. Secondly, the active participation of supporters can guarantee the successful completion of crowdfunding by the project initiator and also enhance the confidence of farmers to carry out agricultural production. Third, the active participation of backers can speed up the timely delivery of products by project initiator, so that backers can enjoy fresh produce as soon as possible. Therefore, for agricultural products crowdfunding, especially those with strong seasonality, the active participation of backers is especially important.

To improve the financing performance and project success rate of crowdfunding, the research on backer behavior in crowdfunding has attracted the attention of scholars, which mainly focuses on three main areas. First, some studies have explored the dynamic process of group behavior of backers in the investment process [7–9]. Second, the impact of the number of likes, views, and comments on the project by backers on financing performance in crowdfunding has also attracted the attention of scholars [10]. Third, studies on the motivations of backers to participate in crowdfunding is also growing in richness [11–14]. Few existing studies on crowdfunding backers have addressed the agriculture. The research on the impact of the investment speed, the investor investment intensity, and the number of early investors on financing performance in agri-food crowdfunding, especially by examining the complex impact mechanisms of initiator and project attributes with systems thinking. Therefore, different from previous studies, this paper combines the current situation of agriculture crowdfunding in China, and explores the impact of backer behavior on project financing performance based on the trust perspective and the herd effect theory. Typically, the project initiators in agriculture crowdfunding post the information about their characteristics and project attributes on crowdfunding webpage. The backers mainly make their investment decisions by browsing the information of initiator characteristics and project attribute disclosed on the crowdfunding webpage. Therefore, this paper also analyzes the complex influence mechanism of the core trust factors of initiator characteristics and project attributes in the relationship between backer behavior and financing performance, with a view to improving financing performance by the three parties working together.

This study provides several potential theoretical contributions to the literature. Firstly, it expands the application of trust theory and herding effect in the research of agri-food crowdfunding. Based on the herd effect theory, this paper systematically analyzes the impact of backer investment behaviors, including the investment speed, the investment intensity, the number of early project backers, the number of backer comments, and the number of selfless backers, on project financing performance. Meanwhile, the role mechanisms of core trust factors of initiator characteristics and project attributes in the relationship between backer behaviors affecting project financing performance are explored based on the trust perspective. Secondly, this paper applies systems thinking to study how the information disclosed in the three aspects of backer behavior, initiator characteristics, and project attributes work together to improve the financing performance of a project in agri-food crowdfunding. As an important participant, backers are an important factor affecting the financing performance. And previous studies mainly select evaluation indicators from three aspects simultaneously, namely, initiator characteristics, project attributes, and backer behaviors, while examining the impact of each of these three factors on project financing performance [15]. Moreover, the evaluation indicators in terms of backer behavior mainly consider backers’ likes and commenting behaviors [16]. There is a lack of research on the mechanism of linkage influence of backer behavior on project financing performance by combining the core trust factors of initiator characteristics and project attributes, which is specifically targeted at backer behavior. However, the effect of disclosed information of the initiator characteristics and project attributes should be demonstrated by the ability to reduce the risk of information asymmetry in crowdfunding, which in turn promotes backer investment and improves financing performance. Therefore, this paper focuses on backers in agriculture crowdfunding, and comprehensively considers the mechanism of the core trust factors of initiator characteristics and project attributes in the relationship between backer behavior and project financing performance. Considering the linkage effect of the disclosed information about initiator, project and backer on project financing performance not only solves the problem of information asymmetry, but also provides a new research idea for exploring the influence mechanism of backer behavior on project financing performance.

This article is structured as follows. First, I review the relevant literature. Then, theoretical background and hypothesis development are developed. Third, I present the research design, including the setting of the data and variables, and the construction of the empirical model. Then, I report the empirical results and test the robustness of the empirical model. Finally, I offer the conclusion based on the empirical results and discuss the contributions and limitations.

## Theory and hypotheses

### Theoretical background

Presently, studies on the impact of backer behavior on financing performance mainly involve the level of attention, likes, views, comments, and number of supporters, etc. The results from Davis et al. [17] shows that perceived product creativity is positively related to crowdfunding financing performance, both directly and indirectly, via positive affective reactions of potential backers. Where backers and experts disagree, it is far more likely to be a case where the backers are willing to invest projects that experts may not [18]. The attention and praise from backers on the crowdfunding platform can, to a certain extent, reflect their love and recognition of the project. Potential backers will judge the quality of a project based on the amount of likes and attention it receives. Crowdfunding projects that have a higher profile generally have more backers, which increases the likelihood of success. Generally, a project with many comments will have many backers, which can effectively increase the success rate of crowdfunding financing. Moutinho and Leite [19] studied the transaction data of crowdfunding projects on the Kickstarter platform and found a significant positive correlation between the number of backer comments and the success rate of project funding. The number of backers at the early stage of a project affects the attention and recognition of potential backers, which in turn affects the success of the project. Studies by Mollick [20] suggest that the number of backers is a key factor influencing the success of crowdfunding. Detailed comments from backers can fully demonstrate the project’s shortcomings, thus reducing the project’s information asymmetry. Wang et al. [21] found that the word count of backer comments on the project had a positive impact on the project success. As a result, scholars agree that the number of backer comments is an indicator of the number of people following the project and the level of interest. A project with more backer comments will receive more attention and are more likely to receive backer investment.

The impact of the investment speed, the amount of investment, the number of early backers, the degree of product innovativeness, the new ventures’ environmental sustainability orientation, and the number of selfless backers on financing performance in crowdfunding has not been addressed by scholars [6, 22–24]. It is clear that the disclosure of initiator characteristics and project information is a key to promoting the active participation of backers in crowdfunding projects. There is a herding effect in crowdfunding with a diminishing marginal effect, i.e., the herding effect is significant in the early stage of crowdfunding and diminishes in the middle and late stages [25]. Mollick [26] found that the number of early backers can accelerate the investment decision of potential backers in crowdfunding, creating the phenomenon of follow-the-leader investment and thus accelerating financing progress. As a result, there is a clear herding effect for projects that have a fast pace of financing in the early stage. Therefore, it is important to apply systems thinking to explore the joint role of backer behavior, initiator characteristics, and project attributes to improve the project financing performance from the perspective of trust, which has been neglected in prior studies. Here, combining the current situation of agriculture crowdfunding in China and the herd effect theory, this paper investigates the effects of five factors on the project financing performance: investment speed, investment intensity, number of early project backers, number of backer comments, and number of disinterested backers; and also examines the complex influence mechanisms of initiators characteristics and core trust factors of project attributes in the relationship between the backer behavior and the financing performance.

**Fig 1:**
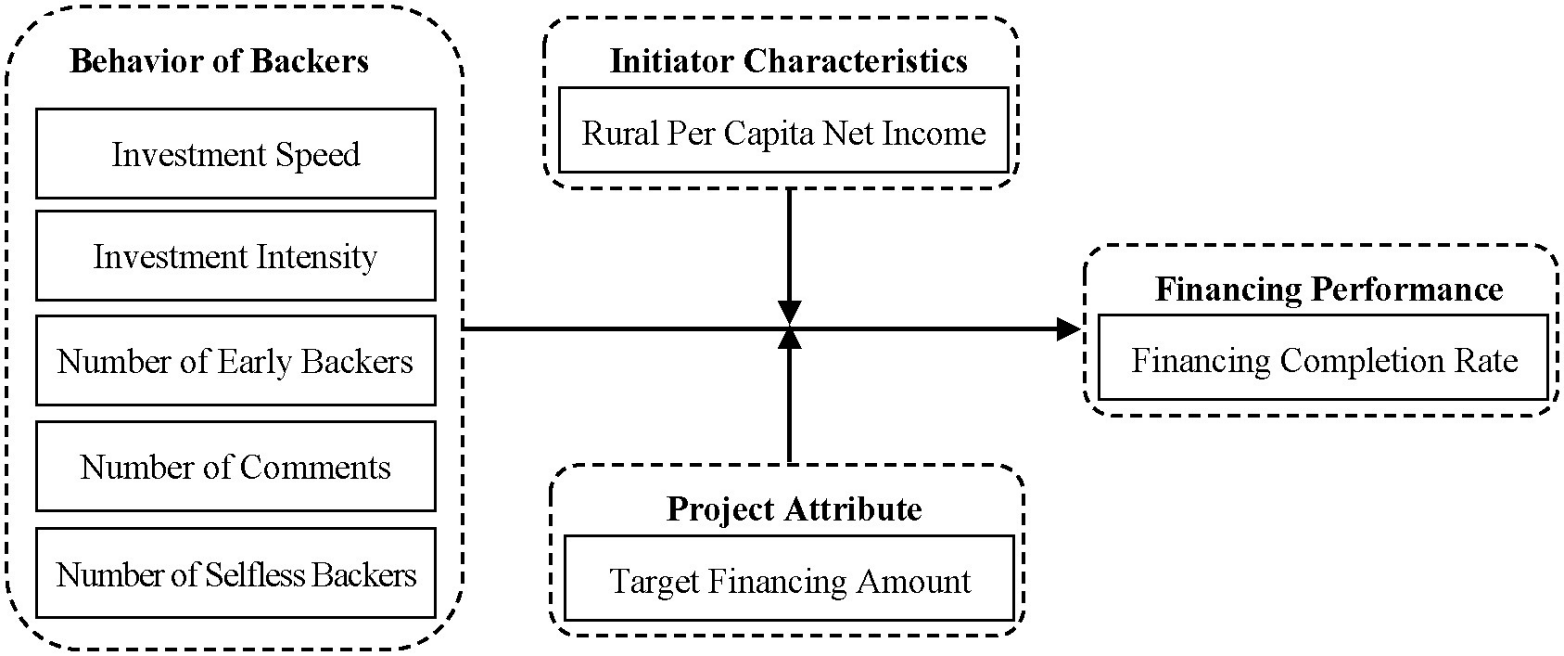
Research model on the mechanism of backer behavior affecting financing performance.

Most of the products in agri-food crowdfunding are unprocessed fresh food products, which require the initiator to achieve the financing goal in the shortest possible time to start the production and sales of the products in time. Attracting more backers in the early stage of project financing can shorten the time to complete the target financing amount, which can help to expand the project financing scale and improve the project financing performance. Clearly, to attract active participation of backers, the initiator should improve the quality of disclosure of information on initiator characteristics and project attributes. So, combining the current situation of agriculture crowdfunding in China and the herding effect theory, this paper investigates both the influence of backer behavior on project financing performance and the moderating role of core trust factors of initiator characteristics and project attributes in the relationship between backer behavior and financing performance. The research model is constructed as shown in Fig 1.

### Hypothesis development

#### Backer behavior and financing performance

*The relationship between the investment speed and investment intensity of backers and the financing performance*. Major crowdfunding platforms commonly implement the “All-or-Nothing (AON)” model, in which initiators that reach their financing target within the preset crowdfunding window can obtain money from backers, or else they will need to return the money to the backers [27, 28]. Hence, how to reach their funding goals within the preset funding period becomes a crucial question for entrepreneurs and startups to ensure crowdfunding success. In the early stage of crowdfunding, the investment behavior of backers shows a herd effect, which can be used to promote potential backers to make investment decisions as soon as possible in order to bring more subsequent investment. The positive promotion effect of financing completion rate on financing performance will show a decreasing marginal effect over time [25, 29]. Therefore, the early completion of the financing target of the project allows for a longer remaining time, thus attracting more subsequent investments. Most of the products in agri-food crowdfunding are perishable and fragile fresh food (such as vegetables, aquatic products, eggs, meat, etc.) with strong randomness in output and demand. Only by increasing the speed and intensity of investment of backers can we ensure that the financing target can be quickly achieved in order to complete the project in the shortest time. Hence, I hypothesize the following:

Hypothesis 1 (H1): The investment speed of backer positively affects the financing performance of a crowdfunding project.
Hypothesis 2 (H2): The investment intensity of backer positively affects the financing performance of a crowdfunding project.

*The relationship between the number of early backers and the financing performance*. Crowdfunding is thus being increasingly used as a marketing tool, rather than a source of capital [30]. The number of early backers of a crowdfunding project is an important indicator of the market acceptance of the product [31]. The serious information asymmetry in the early stage of a project makes the backers tend to follow the investment, which results in a herding effect. Burtch et al. found that reducing access to information controls induces a net increase in fundraising [32, 33]. Previous studies find that a critical factor in crowdfunding success is the early momentum. In recent years, a number of studies discovered the herd effect in crowdfunding, showing that early backers’ influence indeed exists. In contrast, Ma et al. empirically found that early backers could exert not only a positive but also a negative influence on the success of crowdfunding [34]. Potential backers are more likely to pledge in the first and last weeks as compared to the middle period of the funding cycle. There is a strong deadline effect in which project support tends to increase in the last week of the project-funding cycle. Potential backers are less likely to contribute once a project reaches its goal [29]. Different from other crowdfunding, agri-food crowdfunding is essentially a pre-sale of agricultural products. The backers in agri-food crowdfunding are both buyers and consumers of agricultural products. The early backers will make rational investment decisions about whether or not to buy based on their actual need for agricultural products [22]. Supporting the project is also for the purchase of agricultural products. Since the production and sale of agricultural products have high timeliness requirements, the initiators of agri-food crowdfunding projects will increase the number of early backers by designing project plans to ensure the early realization of financing goals, so as to put agricultural products into production as soon as possible and provide fresh agricultural products to backers. When the financing goals of project are achieved earlier, subsequent backers will not hesitate to contribute money to purchase the agricultural products that are determined to be produced. Hence, I hypothesize the following:

Hypothesis 3 (H3): The number of early backers positively affects the financing performance of a crowdfunding project.

*The relationship between the number of backers’ comments and the financing performance*. The number of comments from potential backers in reward-based crowdfunding can reflect the visibility and brand recognition of a project. Analysis based on the herding effect theory found that the number of comments has a positive impact on the financing performance [35]. Based on the analysis of Kickstarter platform data, Mollick found that crowdfunding projects with more followers would have a large number of backers in formal funding, thus increasing the likelihood of project success [20]. Agri-food crowdfunding is actually the pre-sale of agricultural products, and word-of-mouth is very important for agri-food crowdfunding projects. The number of comments has a greater impact on a project’s financing performance than factors in the product’s functionality. Regardless of whether the reviews are positive or negative, projects with a high number of comments tend to be funded by more backers and achieve better financing performance. Hence, I hypothesize the following:

Hypothesis 4 (H4): The number of backers’ comments positively affects the financing performance of a crowdfunding project.

*The relationship between the number of selfless backers and financing performance*. Selfless backers are an important category of investors in crowdfunding. The participation of selfless backers enhances the attention of crowdfunding projects and helps to form a herding effect to attract more backers and improve the success rate [36]. Using Jingdong crowdfunding data as the research samples, Tang [37] found that projects with more selfless backers had a high success rate. Therefore, many projects have set reward types for selfless backers. Agricultural crowdfunding started late in China. The emergence of selfless backers can enhance the popularity and recognition of agri-food crowdfunding projects, eliminate backers’ concerns, and develop markets for agricultural products from remote and poor areas. The herding effect brought by selfless backers can attract more investment from physical investors, thus expanding the scale of financing and improving project financing performance. Hence, I hypothesize the following:

Hypothesis 5 (H5): The number of selfless backers positively affects the financing performance of a crowdfunding project.

#### The mediation role of information of initiator characteristics

To launch a crowdfunding project, initiators must register on the platform and provide all the necessary information related to the project and the company, including their webpage and digital media. Personal characteristics, such as experience, competences, knowledge, gender and formal education, of the entrepreneur are seen as relevant drivers of (young) firm performance and can enhance backers’ trust [38]. It has been widely recognized that initiators’ regional social capital has an important impact on the performance of reward-based crowdfunding. The emergency of Internet has broken the limitations on financing performance imposed by the geographical distance between initiators and backers [39]. Some scholars also argue that Internet-based crowdfunding models have not changed the negative impact of geographic distance on financing performance. Regional preferences still exist, and local projects will still receive more support. Especially for agri-food crowdfunding, the geographical environment of the initiators has a significant impact on the success of the project. A good level of economic development in the initiator’s location can enhance the confidence of backers in the success of the project. Meanwhile, the backers in agri-food crowdfunding are mostly consumers of agricultural products. In order to protect investment security, the backers prefer to invest in projects that are geographically close to them. Additionally, backers in places with good economic development are able to put up more money to support crowdfunding projects. Choosing a nearby agri-food crowdfunding project can also ensure that the produced agricultural products reach the backers in a timely manner. Therefore, this paper uses the net rural income per capita of the initiator’s location as an indicator of the regional economic environment. Hence, I hypothesize the following:

Hypothesis 6 (H6): The rural per capita net income in the location of the initiator positively moderates the influence of backer behavior on project financing performance.
Hypothesis 6–1 (H6-1): The rural per capita net income in the location of the initiator positively moderates the influence of investment speed of backers on project financing performance.
Hypothesis 6–2 (H6-2): The rural per capita net income in the location of the initiator positively moderates the influence of investment intensity of backers on project financing performance.
Hypothesis 6–3 (H6-3): The rural per capita net income in the location of the initiator positively moderates the influence of number of early backers on project financing performance.
Hypothesis 6–4 (H6-4): The rural per capita net income in the location of the initiator positively moderates the influence of number of backers’ comments on project financing performance.
Hypothesis 6–5 (H6-5): The rural per capita net income in the location of the initiator positively moderates the influence of number of selfless backers on project financing performance.

#### The moderate role of project attributes

The "all-or-nothing" model used in reward-based crowdfunding requires projects to set a fundraising window and a target funding amount [27, 28]. Therefore, the target financing amount is an important characteristic information that must be predetermined by each project, and is regarded as the core trust factor of crowdfunding projects. When designing a project proposal, the initiator may set a higher target funding amount in order to obtain more funds, or may set a lower target funding amount for fear of too high a level, which may lead to the failure of the project. Therefore, there is no uniform conclusion on the role of target financing amount in crowdfunding. Usually, initiators demonstrate their strength by setting a high target financing amount to enhance backers’ confidence in the success of the project [40]. However, Ahlers et al. [41] showed that crowdfunding projects with small target financing amounts have high financing success rates. Setting too large a target financing amount can lead to an increased perception of risk for backers, which reduces the likelihood that they will invest. Agricultural production is susceptible to natural conditions, with many uncontrollable factors and high risks. Therefore, setting a lower target financing amount can help improve the success rate of agri-food crowdfunding projects, reduce the uncertain risk of backers, and stimulate backers to contribute more and faster. At the same time, the lower risk perception can also attract more backers to pay attention to the project, participate in project comments, give selfless support to the project, and then attract more early backers to invest. Hence, I hypothesize the following:

Hypothesis 7 (H7): The target financing amount negatively moderates the influence of backer behavior on project financing performance.
Hypothesis 7–1 (H7-1): The target financing amount negatively moderates the influence of investment speed of backers on project financing performance.
Hypothesis 7–2 (H7-2): The target financing amount negatively moderates the influence of investment intensity of backers on project financing performance.
Hypothesis 7–3 (H7-3): The target financing amount negatively moderates the influence of number of early backers on project financing performance.
Hypothesis 7–4 (H7-4): The target financing amount negatively moderates the influence of number of backers’ comments on project financing performance.
Hypothesis 7–5 (H7-5): The target financing amount negatively moderates the influence of number of selfless backers on project financing performance.

## Data and variables

### Data collection

Launched in February 2013, Zhongchou is the earliest and most representative crowdfunding platform in China. Zhongchou is also the largest platform for agri-food crowdfunding in terms of the number of campaigns launched and amount of funds raised. By the end of February 2018, there are 6,584 crowdfunding projects achieve their target financing amount on the Zhongchou platform, including 1,172 agricultural projects. Data were collected on 1,172 campaigns launched in the platform’s agri-food category from October 31, 2013, to February 28, 2018. Of these campaigns, 1,144 had complete information and were included in the study’s sample. The data of each variable were obtained using the following methods: (1) the data on the rural per capita net income were collected from the China Statistical Yearbook, published by the National Bureau of Statistics; (2) the data of three variables of the investment speed, number of early backers, and investment amount were extracted and calculated, item by item, from the details displayed for each project on the Zhongchou platform; (3) the data of other variables were collected through the relevant qualification information disclosed by the project initiator on the Zhongchou platform.

### Definition of variables

Due to the large difference in the target financing amount between projects, the dependent variable, financing performance, was measured by the financing completion rate in this study. The financing completion rate was defined as the ratio of the actual financing amount to the target financing amount. A large financing completion rate indicates that the actual financing amount exceeds the target financing amount and the financing performance is higher. The variables that reflected the investment behavior of backers were selected as the independent variables, including the investment speed, investment intensity, number of early backers, backers’ comments, and number of selfless backers. The variables reflecting information about the initiators’ characteristics and the project attributes were used as control variables. In addition, the control variables were the number of progress updates, number of reward types, availability of a lottery, number of images, duration of videos, and initiator type. Table 1 presents the definition of all variables in this study.

**Table 1:** Description of variables and values assigned.

Variables		Symbol	Description
Dependent variable	Financing completion rate	*FCR*	The actual financing amount for a single project is divided by the target financing amount. (%)
Independent variables	Investment speed	*IS*	Number of days of funding per backer for a single project.
	Investment intensity	*II*	Actual funding success rate divided by the size of the crowd.
	Number of early backers	*NEB*	Total cumulative number of backers in the first two days of a single project’s launch.
	Backers’ comments	*BC*	Number of comments (per campaign) published on the platform by the crowd
	Number of selfless backers	*NSB*	Number of people who donated to a project
Mediating Variables	Rural per capita net income	*RPCI*	The per capita net income of farmers in the region where the initiator is located (in CNY).
	Target financing amount	*TFA*	The amount of money that the project initiator seeks to raise (in CNY).
Control Variables	Number of progress updates	*NPU*	Number of project progress updates published on the platform by the initiator
	Number of reward types	*NRT*	Number of reward types for a single project
	Availability of a lottery	*AL*	Availability of a lottery in the campaign design (yes = 1, no = 0)
	Number of images	*NI*	Number of images initiators use to show the project content on a crowdfunding platform
	Duration of videos	*DV*	Duration (seconds) of videos used in the campaign profile (0 for profiles with no videos)
	Initiator type	*IT*	Dummy = 1 if initiator is an institution initiator; 0 otherwise

### Empirical model

This study used multiple linear regression analysis to investigate the effects of each variable of backer behavior on project financing performance and to examine the moderating effects of the core trust factors of initiator characteristics and project attributes in this context. The following models are used to test my hypotheses.

Model 1 examines the effects of the six control variables on the financing completion rate: number of progress updates (NPU), number of reward types (NRT), availability of a lottery (AL), number of images (NI), duration of videos (DV), and initiator type (IT). Model 1 is expressed as follows.


$$FCR=\alpha_{0}+\alpha_{1}NPU+\alpha_{2}NRT+\alpha_{3}AL+\alpha_{4}NI+\alpha_{5}DV+\alpha_{6}IT+\varepsilon_{1}$$ (1)

Model 2 is constructed by adding five independent variables to Model 1: investment speed (IS), investment intensity (II), number of early backers (NEB), backers’ comments (BC), and number of selfless backers (NSB). The joint effects of the independent variables and the control variables on the financing completion rate were examined. Model 2 is expressed as follows.


$$\begin{array}{ll} FCR= & \beta_{0}+\beta_{1}NPU+\beta_{2}NRT+\beta_{3}AL+\beta_{4}NI+\beta_{5}DV+\beta_{6}IT\\ & +\beta_{7}IS+\beta_{8}II+\beta_{9}NEB+\beta_{10}BC+\beta_{11}NSB+\varepsilon_{2} \end{array}$$ (2)

To test the effect of the moderating variable, rural per capita net income (RPCI), on the relationship between the five independent variables (investment speed, investment intensity, number of early backers, backers’ comments, and number of selfless backers) and project financing performance, Models 3 to 7 were constructed respectively. The five independent variables were standardized before forming an interaction term with rural per capita net income (RPCI). Models 3 to 7 are expressed as follows.


$$\begin{array}{ll} FCR= & \chi_{0}+\chi_{1}NPU+\chi_{2}NRT+\chi_{3}AL+\chi_{4}NI+\chi_{5}DV+\chi_{6}IT+\chi_{7}IS\\ & +\chi_{8}II+\chi_{9}NEB+\chi_{10}BC+\chi_{11}NSB+\chi_{12}RPCI+\chi_{13}RPCI\times IS+\varepsilon_{3} \end{array}$$ (3)

$$\begin{array}{ll} FCR= & \delta_{0}+\delta_{1}NPU+\delta_{2}NRT+\delta_{3}AL+\delta_{4}NI+\delta_{5}DV+\delta_{6}IT+\delta_{7}IS\\ & +\delta_{8}II+\delta_{9}NEB+\delta_{10}BC+\delta_{11}NSB+\delta_{12}RPCI+\delta_{13}RPCI\times IA+\varepsilon_{4} \end{array}$$ (4)

$$\begin{array}{ll} FCR= & \varphi_{0}+\varphi_{1}NPU+\varphi_{2}NRT+\varphi_{3}AL+\varphi_{4}NI+\varphi_{5}DV+\varphi_{6}IT+\varphi_{7}IS\\ & +\varphi_{8}II+\varphi_{9}NEB+\varphi_{10}BC+\varphi_{11}NSB+\varphi_{12}RPCI+\varphi_{13}RPCI\times NEB+\varepsilon_{5} \end{array}$$ (5)

$$\begin{array}{ll} FCR= & \phi_{0}+\phi_{1}NPU+\phi_{2}NRT+\phi_{3}AL+\phi_{4}NI+\phi_{5}DV+\phi_{6}IT+\phi_{7}IS\\ & +\phi_{8}II+\phi_{9}NEB+\phi_{10}BC+\phi_{11}NSB+\phi_{12}RPCI+\phi_{13}RPCI\times NSB+\varepsilon_{6} \end{array}$$ (6)

$$\begin{array}{ll} FCR= & \gamma_{0}+\gamma_{1}NPU+\gamma_{2}NRT+\gamma_{3}AL+\gamma_{4}NI+\gamma_{5}DV+\gamma_{6}IT+\gamma_{7}IS\\ & +\gamma_{8}II+\gamma_{9}NEB+\gamma_{10}BC+\gamma_{11}NSB+\gamma_{12}RPCI+\gamma_{13}RPCI\times BC+\varepsilon_{7} \end{array}$$ (7)

To test the effect of the moderating variable, target financing amount (TFA), on the relationship between the five independent variables (investment speed, investment intensity, number of early backers, backers’ comments, and number of selfless backers) and project financing performance, Models 8 to 12 were constructed respectively. The five independent variables were standardized before forming an interaction term with target financing amount (TFA). Models 8 to 12 are expressed as follows.


$$\begin{array}{ll} FCR= & \eta_{0}+\eta_{1}NPU+\eta_{2}NRT+\eta_{3}AL+\eta_{4}NI+\eta_{5}DV+\eta_{6}IT+\eta_{7}IS\\ & +\eta_{8}II+\eta_{9}NEB+\eta_{10}BC+\eta_{11}NSB+\eta_{12}TFA+\eta_{13}TFA\times IS+\varepsilon_{8} \end{array}$$ (8)

$$\begin{array}{ll} FCR= & \kappa_{0}+\kappa_{1}NPU+\kappa_{2}NRT+\kappa_{3}AL+\kappa_{4}NI+\kappa_{5}DV+\kappa_{6}IT+\kappa_{7}IS\\ & +\kappa_{8}II+\kappa_{9}NEB+\kappa_{10}BC+\kappa_{11}NSB+\kappa_{12}TFA+\kappa_{13}TFA\times IA+\varepsilon_{9} \end{array}$$ (9)

$$\begin{array}{ll} FCR= & \lambda_{0}+\lambda_{1}NPU+\lambda_{2}NRT+\lambda_{3}AL+\lambda_{4}NI+\lambda_{5}DV+\lambda_{6}IT+\lambda_{7}IS\\ & +\lambda_{8}II+\lambda_{9}NEB+\lambda_{10}BC+\lambda_{11}NSB+\lambda_{12}TFA+\lambda_{13}TFA\times NEB+\varepsilon_{10} \end{array}$$ (10)

$$\begin{array}{ll} FCR= & \mu_{0}+\mu_{1}NPU+\mu_{2}NRT+\mu_{3}AL+\mu_{4}NI+\mu_{5}DV+\mu_{6}IT+\mu_{7}IS\\ & +\mu_{8}II+\mu_{9}NEB+\mu_{10}BC+\mu_{11}NSB+\mu_{12}TFA+\mu_{13}TFA\times BC+\varepsilon_{11} \end{array}$$ (11)

$$\begin{array}{ll} FCR= & \nu_{0}+\nu_{1}NPU+\nu_{2}NRT+\nu_{3}AL+\nu_{4}NI+\nu_{5}DV+\nu_{6}IT+\nu_{7}IS\\ & +\nu_{8}II+\nu_{9}NEB+\nu_{10}BC+\nu_{11}NSB+\nu_{12}TFA+\nu_{13}TFA\times NSB+\varepsilon_{12} \end{array}$$ (12)

## Results

### Descriptive statistics

A descriptive analysis of the sample of 1144 projects was performed using IBM SPSS Statistics 22. Table 2 shows the results of the descriptive statistics. The average financing completion rate for each project ranged from a minimum of 100% to a maximum of 5,397%; the average financing completion rate for all the samples was 163%, indicating that the financing completion rate of individual projects varied widely and the financing completion rate of the projects was generally not high. The rural per capita net income (RPCI) in the initiator’s location ranged from a minimum of 6,936.2 yuan to a maximum of 23,205.2 yuan, with an average of 12,521.2 yuan. Analysis of the samples data reveals that the initiators in agri-food crowdfunding mainly come from regions with a relatively good level of agricultural economic development. In addition, the project target financing amount ranged from a minimum of 500 Yuan to a maximum of 500,000 Yuan, with an average of 17,928 Yuan, indicating a wide range of target financing amount among projects, with most projects having a target financing amount of around 20,000 Yuan.

**Table 2:** Descriptive statistics of the sample (N = 1144).

Variable	Minimum	Maximum	Mean	Variance
FCR	100.00	5397.00	163.31	234.88
IS	1.00	70.01	13.5731	10.16
II	0.09	113.67	3.6532	5.24
NEB	1.00	520.00	29.5105	50.39
BC	0.00	312.00	26.66	31.16
NSB	0.00	325.00	8.4773	19.24
RPCI	6936.20	23205.20	12521.20	4212.11
TFA	500.00	500000.00	17928.20	37484.48
NPU	0.00	94.00	5.97	5.37
NRT	1.00	14.00	5.16	1.92
AL	0.00	1.00	0.42	0.49
NI	1.00	107.00	19.89	10.77
DV	0.00	1804.00	58.44	176.03
IT	0.00	1.00	0.30	0.46

To reveal the influence of each variable of backers’ behavior on the project financing completion rate, an internal structural analysis of the backers’ behavior variables was conducted, and the results are shown in Table 3.

**Table 3:** Internal structural analysis of variables.

Variable	Category	Number of Projects	Proportion (%)
IS	1–7	329	28.76
	8–14	389	34.00
	15–21	228	19.93
	≥22	198	17.31
II	<5%	892	77.97
	≥5%	980	22.03
NEB	<10	491	42.92
	≥10	653	57.08
BC	Yes	1128	98.6
	No	16	1.4
NSB	<50	957	83.65
	≥50	187	16.35

As shown in Table 3, the backers’ investment speed in 28.76% of the projects was less than 7 days and the backers’ investment speed in 63.76% was less than 14 days, indicating that backers funded within the first two weeks on average. There are 892 projects with backers’ investment intensity less than 5%, accounting for 77.97%, indicating that backers’ investment is generally not large and the investment intensity needs to be increased. A total of 57.08% of the projects had early backers of more than 10, indicating that more than half of the projects have a high number of early backers. A total of 1128 projects had potential backers participating in the comments, accounting for 98.6%, indicating that potential backers were more active in participating in comments. A total of 83.65% of projects had selfless backers, indicating that agricultural crowdfunding has a large number of selfless participants.

### Empirical analysis

#### Correlation between variables

Prior to the regression analysis, Pearson correlation analysis was conducted for each variable, as shown in Table 4. The results show that the two variables with the highest correlation coefficients are the number of early backers and the investment intensity, and the correlation coefficient between them is 0.555 (˂0.8), while the correlation coefficients between all other variables are less than 0.5, indicating that there is no strong correlation between the variables. According to the results of the regression equation variance analysis, the significance of the test values is less than 0.001, indicating that the equation is highly significant; that is, the null hypothesis that all coefficients are zero is rejected.

**Table 4:** Correlation coefficients between variables.

	FCR	IS	II	NEB	BC	NSB	RPCI	TFA	NPU	NRT	AL	NI	DV	IT
FCR	1													
IS	-0.043	1												
II	0.296**	-0.062*	1											
NEB	0.131**	-0.412**	-0.558**	1										
BC	0.239**	0.144**	-0.448**	0.337**	1									
NSB	0.001	-0.072*	-0.446**	0.380**	0.119**	1								
RPCI	0.042	-0.039	-0.075*	0.034	0.105**	-0.085**	1							
TFA	-0.183**	0.123**	-0.618**	0.297**	0.450**	0.258**	0.194**	1						
NPU	0.031	0.113**	-0.242**	0.145**	0.426**	0.041	-0.038	0.130**	1					
NRT	0.000	0.048	-0.068*	0.058	0.143**	-0.053	0.007	0.084**	0.143**	1				
AL	-0.026	-0.009	-0.266**	0.230**	0.061*	0.038	0.059*	0.024	0.120**	0.263**	1			
NI	0.063*	-0.017	-0.001	0.026	0.084**	0.040	0.026	.053	.077**	.250**	.062*	1		
DV	0.001	-0.041	-0.069*	0.079**	0.064*	0.041	0.001	.101**	.023	.031	.086**	.105**	1	
IT	-0.081**	-0.058*	-0.190**	0.151**	0.082**	0.158**	0.015	.151**	.073*	.020	.077**	.072*	.026	1

Note

* Significant at 5%.

** Significant at 1%.

Additionally, in order to determine whether there was multicollinearity problem among the variables, the variance inflation factor test was performed before regression analysis. As shown in Table 5, the VIF values of the variables in the model were all less than 3, indicating that there is no multicollinearity problem among the variables.

**Table 5:** Multicollinearity test.

Variable	Tolerance	VIF
IS	0.655	1.527
II	0.360	2.778
NEB	0.439	2.276
BC	0.586	1.707
NSB	0.729	1.371
RPCI	0.922	1.085
TFA	0.521	1.919
NPU	0.781	1.281
NRT	0.847	1.181
AL	0.796	1.256
NI	0.911	1.098
DV	0.969	1.032
IT	0.943	1.060

#### Regression analysis

Multiple regression analysis was performed to test the effects of independent and moderating variables on the dependent variable after classifying the variables. Table 6 shows the results of the main effects regression analysis. Model 1 tests the effect of control variables on the dependent variable (financing completion rate). Compared with Model 1, Model 2 adds independent variables reflecting the behavior of backers to test the effect of control variables and independent variables together on the dependent variable. To assess the probability changes associated with each variable, the coefficients in the table are non-standardized.

**Table 6:** Main effect multiple regression results of research models.

Variable	Financing completion rate	
	Model 1	Model 2
*Control variables*		
*NPU*	0.025	-0.031*
	(0.213)	(0.072)
*NRT*	-0.018	-0.061**
	(0.641)	(0.042)
*AL*	-0.033	0.112***
	(0.437)	(0.001)
*NI*	0.061**	0.023
	(0.020)	(0.261)
*DV*	-0.001	-0.003
	(0.916)	(0.572)
*IT*	-0.129***	-0.073**
	(0.003)	(0.038)
*Independent variables*		
*IS*		-0.088***
		(0.000)
*II*		0.359***
		(0.000)
*NEB*		0.155***
		(0.000)
*BC*		0.204***
		(0.000)
*NSB*		0.062***
		(0.000)
F	2.565**	68.201***
	(0.018)	(0.000)
R^2^	0.013	0.399
Adjusted R^2^	0.008	0.393

Note:

* Significant at 10%.

** Significant at 5%.

*** Significant at 1%.

The regression results of Model 2 show that investment speed has a negative effect on the financing completion rate at the 1% level. This indicates that backers’ fast funding contributes to the financing performance of agri-food crowdfunding projects. Thus, H1 is supported. The four independent variables of investment intensity, number of early backers, number of backers’ comments and number of selfless backers have a significant positive effect on the financing completion rate at 1% level, which indicates that agri-food crowdfunding projects can enhance financing performance by promoting backers to invest more and attracting more attention and investment from early backers and selfless backers. Thus, H2, H3, H4 and H5 are supported.

#### The moderating effect of initiator characteristics

To investigate the role of initiator characteristics in the impact of backers’ behavior on the financing performance, the rural per capita net income, the core trust factor of initiator characteristics, is taken as a moderating variable, and the cross-product term between the rural per capita net income and the relevant measurement indicators of backers’ behavior is added to construct Model 3, Model 4, Model 5, Model 6 and Model 7. The regression results are shown in Table 7.

**Table 7:** Regression results of moderating effect of rural per capita net income.

Variable	Financing completion rate				
	Model 3	Model 4	Model 5	Model 6	Model 7
Control variables					
NPU	-0.029*	-0.029**	-0.023	-0.026	-0.028
	(0.098)	(0.091)	(0.178)	(0.128)	(0.107)
NRT	-0.056*	-0.060**	-0.057*	-0.058*	-0.065**
	(0.063)	(0.046)	(0.053)	(0.053)	(0.031)
AL	0.114***	0.104***	0.116***	0.108**	0.102***
	(0.001)	(0.003)	(0.001)	(0.002)	(0.003)
NI	0.024	0.020	0.026	0.022	0.019
	(0.249)	(0.324)	(0.202)	(0.297)	(0.361)
DV	-0.003	-0.004	-0.002	-0.003	-0.002
	(0.582)	(0.483)	(0.669)	(0.603)	(0.727)
IT	-0.076**	-0.069**	-0.071**	-0.073**	-0.069**
	(0.032)	(0.049)	(0.040)	(0.038)	(0.049)
Independent variables					
IS	0.901*	0.092***	0.101***	0.091***	0.094***
	(0.053)	(0.000)	(0.000)	(0.000)	(0.000)
II	0.362***	0.367***	0.373***	0.363***	0.364***
	(0.000)	(0.000)	(0.000)	(0.000)	(0.000)
NEB	0.155***	0.158***	0.103***	0.157***	0.159***
	(0.000)	(0.000)	(0.000)	(0.000)	(0.000)
BC	0.201***	0.201***	0.194***	0.199***	0.201***
	(0.000)	(0.000)	(0.000)	(0.000)	(0.000)
NSB	0.067***	0.065***	0.068***	0.066***	0.062***
	(0.000)	(0.000)	(0.000)	(0.000)	(0.000)
Mediating Variables					
RPCI	0.288*	0.102***	0.074**	0.085**	0.093***
	(0.018)	(0.004)	(0.033)	(0.017)	(0.008)
RPCI*IS	-0.078*				
	(0.082)				
RPCI*II		0.053***			
		(0.001)			
RPCI*NEB			0.000***		
			(0.000)		
RPCI*BC				0.004	
				(0.693)	
RPCI*NSB					-0.033***
					(0.001)
F	58.750***	59.820***	64.889***	58.381***	59.815***
	(0.000)	(0.000)	(0.000)	(0.000)	(0.000)
R^2^	0.403	0.408	0.427	0.402	0.408
Adjusted R^2^	0.396	0.401	0.421	0.395	0.401

Note:

* Significant at 10%.

** Significant at 5%.

*** Significant at 1%.

Models 3 to 7 test the moderating effect of the rural per capita net income. The regression coefficients of the cross-product terms in Models 3 and 4 are -0.078 at the 10% level and 0.053 at the 1% level, respectively. This suggests that backers invested in projects in areas with high rural per capita net income at a faster rate and with higher intensity, which improves the financing completion rate of projects. The regression results in Model 5 confirm that the rural per capita net income positively moderates the effect of the number of early backers on the financing completion rate. The significance level of the regression coefficients of the cross-product term in Model 6 is greater than 10%, indicating that the moderating effect of the rural per capita net income is not significant. The regression coefficient of the cross-product term in Model 7 is -0.033 at the 1% level, which suggests that the rural per capita net income reduces the contribution of selfless backers to the financing completion rate. Thus, H6 is supported. For agri-food crowdfunding, the economic environment of the initiator’s location affects the role of backers’ behavior on the project financing performance. The excellent regional economic environment and the agricultural products with regional characteristics can enhance the interest and the trust perception of backers, thus contributing to the financing performance of agri-food crowdfunding project.

#### The moderating effect of project attributes

In order to investigate the moderating effect of project attribute on the role of backers’ behavior, the target financing amount, the core trust factor of project attribute, is taken as a moderating variable, and the cross-product term between the target financing amount and the relevant measurement indicators of backers’ behavior is added to construct Model 8, Model 9, Model 10, Model 11 and Model 12. The regression results are shown in Table 8.

**Table 8:** Regression results of moderating effect of target financing amount.

Variable	Financing completion rate				
	Model 8	Model 9	Model 10	Model 11	Model 12
Control variables					
NPU	-0.038**	-0.037**	-0.036**	-0.038**	-0.038**
	(0.026)	(0.033)	(0.039)	(0.028)	(0.028)
NRT	-0.054*	-0.055*	-0.054*	-0.055*	-0.060**
	(0.073)	(0.068)	(0.069)	(0.066)	(0.044)
AL	0.094***	0.098***	0.103***	0.092**	0.092***
	(0.008)	(0.006)	(0.004)	(0.010)	(0.009)
NI	0.024	0.029	0.026	0.024	0.027
	(0.254)	(0.154)	(0.214)	(0.237)	(0.194)
DV	-0.002	-0.001	-0.002	-0.002	-0.001
	(0.735)	(0.774)	(0.693)	(0.751)	(0.819)
IT	-0.069**	-0.070**	-0.065*	-0.067*	-0.065*
	(0.050)	(0.047)	(0.064)	(0.056)	(0.065)
Independent variables					
IS	0.089***	0.093***	0.091***	0.090***	0.092***
	(0.000)	(0.000)	(0.000)	(0.000)	(0.000)
II	0.333***	0.324***	0.332***	0.333***	0.336***
	(0.000)	(0.000)	(0.000)	(0.000)	(0.000)
NEB	0.152***	0.153***	0.148***	0.153***	0.153***
	(0.000)	(0.000)	(0.000)	(0.000)	(0.000)
BC	0.215***	0.213***	0.213***	0.215***	0.216***
	(0.000)	(0.000)	(0.000)	(0.000)	(0.000)
NSB	0.062***	0.062***	0.064***	0.062***	0.065***
	(0.000)	(0.000)	(0.000)	(0.000)	(0.000)
Mediating Variables					
TFA	-0.032***	-0.042***	-0.037***	-0.031***	-0.031***
	(0.003)	(0.000)	(0.001)	(0.003)	(0.003)
TFA *IS	-0.015				
	(0.123)				
TFA *II		-0.044**			
		(0.016)			
TFA *NEB			0.024***		
			(0.005)		
TFA *BC				-0.004	
				(0.529)	
TFA *NSB					-0.024**
					(0.047)
F	59.109***	59.545***	59.829***	58.852***	59.311***
	(0.000)	(0.000)	(0.000)	(0.000)	(0.000)
R^2^	0.405	0.407	0.408	0.404	0.406
Adjusted R^2^	0.398	0.400	0.401	0.397	0.399

Note:

* Significant at 10%.

** Significant at 5%.

*** Significant at 1%.

Models 8 to 12 test the moderating effect of target financing amount. Since the significance level of the regression coefficient of the cross-product term in Model 8 is greater than 10%, it indicates that the setting of the target financing amount has no effect on the role of backers’ investment speed on the financing completion rate. The regression coefficient of the cross-product term in Model 9 is -0.044 at the 5% level, indicating that setting the target financing amount too high will reduce the effect of backers’ investment intensity on the financing completion rate. The regression results in Model 10 indicate that the target financing amount positively moderates the effect of the number of early backers on the financing completion rate at the 1% level. In Model 11, the target financing amount does not significantly moderate the effect of the number backers’ comments on the financing completion rate. The regression results of Model 12 indicate that a high target financing amount will reduce the effect of the number of selfless backers on the project financing completion rate. In summary, most of H7 is supported.

## Discussion and implications

### Conclusions

This paper explores the impact mechanism of the backers’ investment behavior on the financing performance from five aspects: investment speed of backer, investment intensity of backer, number of early backers, number of backers’ comments, and number of selfless backers. Combined with the initiator characteristics and project attributes, this paper discusses how the information of project, initiator and backers work together to improve the project financing performance. The empirical test results are shown in Table 9. The research conclusions are as follows: First, the five factors embodying backers’ investment behavior have a significant impact on the financing performance of agri-food crowdfunding projects. Increasing the speed and intensity of backers’ investment, and the number of early backers can improve the financing performance of agri-food crowdfunding projects. Second, the rural per capita net income, an indicator reflecting initiator characteristics, positively moderates the effect of the investment speed, investment intensity, and number of early backers on financing performance, excluding the number of backers’ comments and number of selfless backers. Third, the indicator representing project attributes, target financing amount, partially moderates the effect of backer investment behavior on financing performance. The effects of the investment intensity and number of selfless backers on financing performance are negatively moderated by the target financing amount. The effect of the number of early backers on financing performance is positively moderated by the target financing amount. There is no moderating effect of the target financing amount on the effects of investment speed and number of backers’ comments on project financing performance.

**Table 9:** Summarizes the hypothesis testing results.

Hypothesis		Results
H1	The investment speed of backer positively affects the financing performance of a crowdfunding project.	Support
H2	The investment intensity of backer positively affects the financing performance of a crowdfunding project.	Support
H3	The number of early backers positively affects the financing performance of a crowdfunding project.	Support
H4	The number of backers’ comments positively affects the financing performance of a crowdfunding project.	Support
H5	The number of selfless backers positively affects the financing performance of a crowdfunding project.	Support
H6	The rural per capita net income in the location of the initiator positively moderates the influence of backer behavior on project financing performance.	Partial Support
H6-1	The rural per capita net income in the location of the initiator positively moderates the influence of investment speed of backers on project financing performance.	Support
H6-2	The rural per capita net income in the location of the initiator positively moderates the influence of investment intensity of backers on project financing performance.	Support
H6-3	The rural per capita net income in the location of the initiator positively moderates the influence of number of early backers on project financing performance.	No Support
H6-4	The rural per capita net income in the location of the initiator positively moderates the influence of number of backers’ comments on project financing performance.	No Support
H6-5	The rural per capita net income in the location of the initiator positively moderates the influence of number of selfless backers on project financing performance.	No Support
H7	The target financing amount negatively moderates the influence of backer behavior on project financing performance.	Partial Support
H7-1	The target financing amount negatively moderates the influence of investment speed of backers on project financing performance.	No Support
H7-2	The target financing amount negatively moderates the influence of investment intensity of backers on project financing performance.	Support
H7-3	The target financing amount negatively moderates the influence of number of early backers on project financing performance.	No Support
H7-4	The target financing amount negatively moderates the influence of number of backers’ comments on project financing performance.	No Support
H7-5	The target financing amount negatively moderates the influence of number of selfless backers on project financing performance.	Support

### General discussion

Reward-based crowdfunding is a fast-growing platform in which initiators solicit initial capital from potential backers and offer rewards in exchange. As a special type of reward-based crowdfunding, the financing performance and success rate of agri-food crowdfunding projects have received extensive attention from scholars. Based on the trust theory and the herd effect theory, this study investigates the impact mechanism of the backers’ investment behavior on the financing performance in agri-food crowdfunding, and the moderating effect of the two core trust factors, the initiator characteristics and the project attributes.

First, this study found that the speed and intensity of backers’ investment, and the number of early backers positively affected the financing performance of agri-food crowdfunding projects, which confirmed the existence of the herd effect in crowdfunding. Compared with other reward-based crowdfunding, the product quality in agri-food crowdfunding is highly uncontrollable and the production of products is susceptible to environmental influences. Without being able to judge whether a project is good or bad, potential backers prefer to observe the investment behavior of early backers to make investment decisions. A large number of early backers can increase the likelihood of crowdfunding projects success, which in turn improves the willingness of subsequent backers to investment, creating a herd effect. Agri-food crowdfunding projects with a large number of selfless backers and backers’ comments indicate a high level of attention. Based on the herd effect theory [7, 42], more subsequent backers can be encouraged to invest, thus improving the project’s financing performance.

Second, the indicator reflecting initiator characteristics, the rural per capita net income, has different moderating roles in the effect of various variables of backer behavior on financing performance. It is found that the rural per capita net income, which represents regional social capita, has a significant positive moderating effect on the impacts of three factors, namely, the speed and intensity of backers’ investment, and the number of early backers, on the financing performance of the project, which is consistent with the findings of the previous studies [20, 39]. It indicates that the backers in agri-food crowdfunding trust the projects in regions with good agricultural geographic economic environments. However, special attention should be paid to the fact that the rural per capita net income weakened the effect of the number of selfless backers on project financing performance, which is contrary to the findings of previous studies [43, 44]. It may be because the favorable economic environment in places with high rural per capita net income increases backers’ trust and support for agri-food crowdfunding projects, which in turn enhances project success. In this case, due to the bystander effect, selfless backers may perceive that the project does not need their support and reduce their willingness to support disinterestedly.

Third, the results of this paper show that the indicator reflecting project attributes, target financing amount, negatively moderated the effect of the investment intensity of backers and the number of selfless backers on project financing performance. It indicates that backers in agri-food crowdfunding tend to invest in projects with small target financing amounts. Previous studies on the impact of target financing amount on financing performance have come to different conclusions. Burtch et al. (2013) found that a large target financing amount can reflect the initiator’s confidence in the success of the project, which in turn enhances the backers’ approval of investing in the project [45]. But when it comes to reward crowdfunding, most scholars get the opposite conclusion. Ahlers et al. proved that crowdfunding projects with smaller target financing amount are more likely to be successful [41]. Setting the target financing amount too large leads to the backers’ lack of confidence in the success of the project, which reduces the backers’ willingness to invest. The research conclusion of this paper suggests that the higher the target financing amount of agri-food crowdfunding projects that are still in their infancy in China, the stronger the uncertainty perceived by backers, thus affecting the financing performance and increasing the risk of project failure. Surprisingly, however, the target financing amount positively moderates the effect of the number of early backers on financing performance. It suggests that the early backers in agri-food crowdfunding are more rational and believe that the projects with high target financing amount are of good quality. At the same time, it also shows that the project initiator is well-prepared and confident in the success of the project, which can increase the number of early backers and form a herd effect to drive more investment from subsequent potential backers.

### Implications for practice

In the context of vigorously promoting rural poverty alleviation in China, the development of agri-food crowdfunding can both solve the problem of financing constraints for agricultural development and guide farmers in choosing appropriate agricultural production models. At the same time, agri-food crowdfunding can also be a good solution to the production and sales of agricultural products, which is of great significance to the realization of agricultural industrialized production. The practical implications of this paper are as follows.

Firstly, for the initiator, original and high-quality agricultural products with regional characteristics should be explored for crowdfunding, and the content of the project book should be carefully designed. A reasonable target financing amount should be set to improve the financing performance in agri-food crowdfunding. A detailed explanation of the reasons for crowdfunding should be provided to motivate more selfless backers to invest, thus gathering popularity and attention for the project to attract more followers. In addition, the initiator should communicate and interact with potential backers in a timely manner, pay attention to potential backers’ comments on the project, and respond to questions raised by backers in order to eliminate backers’ doubts and motivate them to invest as soon as possible.

Secondly, for crowdfunding platforms, they should increase the publicity and the social influence in order to attract the attention of backer and create a good atmosphere for agri-food crowdfunding. A platform for communication and interaction between initiators and backers should be constructed to facilitate timely announcement of project information and response to backers’ concerns. Appropriate rewards are given to active initiators and backers. At the same time, the platform should help the initiators to design the project plan, provide a detailed explanation of the reasons for crowdfunding, plan the use of funds and set a reasonable target financing amount.

Thirdly, for regulators, they should widely publicize agri-food crowdfunding and increase its popularity. Farmers should be encouraged to develop high-quality agricultural products with regional characteristics, and to carry out trademark registration and product certification to enhance the credibility of their products. The participation behavior of potential backers should be appropriately restrained and guided. Create an honest environment for crowdfunding transactions to enhance backers’ trust in the online environment, and then increase trust in agri-food crowdfunding. Building a bridge and link for communication and exchange between initiators and consumers to achieve an effective match between urban residents’ demand for green, safe, high quality and personalized agricultural products and the production of original ecological agricultural products in rural areas, so as to attract investment from backers.

### Limitations and future directions

Nevertheless, my research still has some limitations and it can provide opportunities for future research.

Firstly, this paper attempts to clarify the influence mechanism of backer behavioral factors on the financing performance in agricultural crowdfunding, so as to guide the initiators and platforms to better design crowdfunding projects. However, due to the difficulties encountered in data collection, I only considered five backer behavioral factors: the investment speed, the investment intensity, the number of early backers, the backers’ comments, and the number of selfless backers. Whether there are other behavioral factors remains to be further explored. Follow-up studies can dig deeper into the deeper behavioral aspects of backers through questionnaires, in-depth interviews or experiments.

Secondly, the evaluation capability of the research model constructed in this paper is still inadequate. The research model in this paper explains the influence of backers’ behavior factors on the financing performance in agri-food crowdfunding, and also explores the moderating role of the core trust factors of the initiator characteristics and project attribute. However, given the length of the paper, only the moderating mechanisms of the rural per capita net income, which represents the initiator characteristics, and the target financing amount, which represents the project attribute characteristics, are explored in this paper. Subsequent studies can systematically and deeply explore the moderating mechanisms of other factors of sponsor characteristics and project attributes in the study model.

Thirdly, the sample data in this paper is obtained from Zhongchou platform, a professional platform for publishing agricultural crowdfunding projects, excluding sample data of campaigns launched on other platforms, especially agricultural vertical platforms. When more data from other platforms are available, cross-platform research on the behavior of agricultural crowdfunding backers can be conducted. Additionally, as crowdfunding in the agricultural industry develops further, research can be conducted to differentiate the behavior of supporters of different types of agricultural crowdfunding projects, such as agricultural equity crowdfunding, agricultural technology crowdfunding, and farm crowdfunding.
